# Effect of Er, Si, Hf and Nb Additives on the Thermal Stability of Microstructure, Electrical Resistivity and Microhardness of Fine-Grained Aluminum Alloys of Al-0.25%Zr

**DOI:** 10.3390/ma16052114

**Published:** 2023-03-06

**Authors:** Aleksey V. Nokhrin, Galina S. Nagicheva, Vladimir N. Chuvil’deev, Vladimir I. Kopylov, Aleksandr A. Bobrov, Nataliya Yu. Tabachkova

**Affiliations:** 1 Materials Science Department, Physical and Technical Research Institute, Lobachevsky State University of Nizhny Novgorod, 603022 Nizhny Novgorod, Russia; 2 Center Collective Use “Materials Science and Metallurgy”, National University of Science and Technology “MISIS”, 119991 Moscow, Russia; 3 Laboratory “FIANIT”, Laser Materials and Technology Research Center, A.M. Prokhorov General Physics Institute of the Russian Academy of Sciences, 119991 Moscow, Russia

**Keywords:** aluminum alloys, Al-Zr, microstructure, electrical conductivity, microhardness, diffusion

## Abstract

The conductor aluminum alloys of Al-0.25wt.%Zr alloyed additionally with X = Er, Si, Hf and Nb were the objects of our investigations. The fine-grained microstructure in the alloys was formed via equal channel angular pressing and rotary swaging. The thermal stability of the microstructure, specific electrical resistivity and microhardness of the novel conductor aluminum alloys were investigated. The mechanisms of nucleation of the Al_3_(Zr, X) secondary particles during annealing the fine-grained aluminum alloys were determined using the Jones–Mehl–Avrami–Kolmogorov equation. Using the Zener equation, the dependencies of the average secondary particle sizes on the annealing time were obtained on the base of the analysis of the data on the grain growth in the aluminum alloys. The secondary particle nucleation during long-time low-temperature annealing (300 °C, 1000 h) was shown to go preferentially at the cores of the lattice dislocations. The Al-0.25%Zr-0.25%Er-0.20%Hf-0.15%Si alloy subjected to long-time annealing at 300 °C has the optimal combination of microhardness and electrical conductivity (59.8%IACS, Hv = 480 ± 15 MPa).

## 1. Introduction

At present, high-strength aluminum alloys with increased electrical conductivity are considered as substitutes for copper alloys applied widely for making the fine wires (∅0.2–0.5 mm) of the on-board wiring of modern aircraft and automotives. International companies use fine bimetallic wires and copper-clad aluminum wires made from complex aluminum alloys for novel airplane and automotive electrical wiring [1,2]. The fine aluminum wires can also be applied efficiently in the car building industry, electric power engineering, power transmission applications, etc. High strength and electrical conductivity are the key characteristics of the conductor aluminum alloys. With conventional methods of processing commercial purity aluminum or ultra-low-alloying commercial aluminum alloys, high strength and electrical conductivity are hardly compatible [3,4,5,6,7]. One of the approaches to the development of novel conductor aluminum alloys is their alloying with the elements, which affects the electrical conductivity weakly but influences their strength positively. In Russia, the highly alloyed eutectic alloy 01417 has been developed, whereby the fine copper-clad aluminum wires from which are applied extensively in the cable industry at present [8,9,10]. For example, the industrial alloys of 01417 have a minimal ultimate strength of 160 MPa, relative elongation of 8% and specific electrical resistivity (SER) of 3.2 μΩ·cm [8]. At present, novel eutectic aluminum alloys alloyed with Ce and La are being developed extensively [11,12,13,14,15].

Modern automotive and aviation technology imposes more and more high requirements upon the fine wires with regard to strength and thermal stability of the microstructure, which cannot be met using commercial aluminum alloys anymore. In connection, aluminum alloy modifications of Al-Mg-Si system alloys (commercial alloys of 6XXX series [16,17,18,19,20], modified Al-Mg-Si-Zr alloys [21], the novel Al-Mg-Si-Cu-Ni-Fe-Sc-Zr alloy [22], the Al-Mg-Si-Fe-Cu alloy [23], etc.), conductor alloys of Al-Fe [24,25,26] and novel aluminum alloys multi-alloyed with various rare earth elements (REEs) and transition metals (TMs)—zirconium, scandium, hafnium, yttrium, etc.—are being developed extensively [27,28,29,30].

Previously, investigations have demonstrated that the Sc-containing aluminum alloys have high strength and thermal stability characteristics [27,29,30,31,32,33,34,35] but wide application of these ones is limited by the high cost of Al-Sc master alloys. This makes the problem of the development of aluminum alloys economically alloyed with scandium, as well as the development of alloys, whereby expensive scandium is replaced by microadditives of cheaper REE and TM, relevant [36,37,38].

At present, investigations on the development of the conductor alloys in the Al-Zr system are being carried out extensively [39,40,41,42,43,44,45]. The increased temperatures and times of nucleation of Al_3_Zr particles (about 50–100 h at the temperatures over 350–400 °C) are a disadvantage of this system from the viewpoint of the prospects of application as heat-resistant wires. This does not allow for providing the stabilization of nonequilibrium fine-grained microstructures in the wires during long-term operation at ~180–220 °C, whereas preliminary annealing for the nucleation of the secondary particles often reduces the plasticity of the workpieces and limits the opportunity to make the fine wires by rolling at reduced temperatures. To solve this problem, Al-Zr alloys are developed with a zirconium concentration up to 0.4–0.6 wt.%, which are made using special ultrafast crystallization technologies [39,40,42,43,44,45,46]. A more efficient way is the additional microalloying of Al-Zr alloys with chemical elements (most often, Er [27,30,47,48,49,50], Hf [30,34,46], Y [30,51,52,53,54], Yb [55,56,57], etc.) providing the accelerated decomposition of solid solutions at lower annealing temperatures. Note that additional alloying allows for avoiding the nucleation of discontinuous large fan-shaped precipitation, which is observed in Al-Zr alloys often [56,57,58,59,60].

In the present work, we investigated the effect of Er, Hf, Nb and Si additives on the thermal stability of the microstructure in Al-Zr alloys. The choice of Er and Hf was caused by positive effects of those on the acceleration of nucleation of Al_3_Zr secondary particles in Al-Zr alloys [46,48,49,50]. The alloying of aluminum with niobium allows the forming of the Al-Nb particles with a D0_23_ structure and also allows the controlling of the character of distribution of Al_3_Zr particles and the forming of the AlZrNb secondary particles additionally [61]. The choice of Si as an alloying element was caused by its effect on the kinetics of nucleation of secondary particles. Si is capable of accelerating the nucleation of the secondary particles in aluminum alloys of Al–Zr [57,62,63,64]. The highest concentration of zirconium in the alloys investigated is limited to 0.25%Zr since large primary Al_3_Zr particles are formed in the alloy at higher concentrations. These particles can be the sources of fracture of the fine wires of 0.2–0.5 mm in diameter during fabrication via various cold deformation methods (drawing, rolling, etc.). Note also that the formation of the Al_3_Zr particles at higher zirconium concentrations works via discontinuous precipitation of the solid solution mechanism often [56,57,58,59,60]. As a consequence, their contributions into the strength and thermal stability of the aluminum wires decrease.

The present work was aimed at the investigation of the effect of the micro additives of Er, Si, Hf and Nb on the thermal stability of the conductor aluminum alloys of Al-0.25%Zr, whereby the fine-grained microstructure of which was formed by combining the technologies of equal channel angular pressing (ECAP) [65] and rotary swaging [66,67,68]. The application of ECAP and rotary swaging technologies allows eliminating the dendrite nonuniformity of the cast macrostructure of the aluminum alloys as well as forming a uniform fine-grained microstructure in the aluminum alloy. It allows forming the microstructure in the aluminum alloys, the parameters of which are similar to the microstructure of fine bimetallic wires made by rolling, extrusion or drawing [1,2,13,33,34]. It should be stressed here that the formation of the fine-grained microstructure affects the intensity and mechanisms of the nucleation of the secondary particles (Al_3_Sc, Al_3_Zr, etc.) in the aluminum alloys [35] and also affects the mechanical properties and performance characteristics of the aluminum alloys (strength and hardness, fatigue resistance, ductility at room and elevated temperatures, etc.) positively [4,6,7,14,17,18,19].

The applied goal of the work was achieving the SER of 3.0 μΩ·cm or less in the novel conductor alloys whilst simultaneously ensuring a high microhardness of these ones.

## 2. Materials and Methods

The aluminum alloys, the compositions of which are presented in Table 1, were the objects of our investigations.

The workpieces of the aluminum alloys of 20 × 20 × 160 mm^3^ in size were obtained via induction casting using an Indutherm^®^ VTC-200V casting machine (Indutherm GmbH, Walzbachtal, Germany) in a vacuum in the regimes specified in Table 2. To prepare the alloys, aluminum A99 as well as Al-3%Zr, Al-3%Hf, Al-3%Si, Al-3%Er and Al-2%Nb master alloys, obtained via the induction casting followed by rolling into foils of 0.2 mm in thickness, were used.

The workpieces obtained were subjected to N = 4 ECAP cycles at 250 °C. ECAP was performed according to the “A” route using hydraulic press Ficep^®^ HF400L (Ficep^®^ S.P.A., Varese, Italy) in square cross-section hardware with a channel crossing angle of 90°. The strain rate in ECAP was 0.1 mm/s. After ECAP, the samples were subjected to rotary swaging at room temperature (RT) using a machine R5-4-21 HIP manufactured by Heinrich Müller Maschinenfabrik GmbH company (Pforzheim, Germany). The rod samples with initial diameters of 20 mm were subjected to rotary swaging down to the diameters of 6.0 mm. The overall strain of the rods was 70%. The obtained cylindrical samples of 6 mm in diameter and 12 mm long were subjected to sediment up to the strain of 35%. The sediment was performed at RT using a 40-ts hydraulic press EU-40 (Germany).

As a result of the application of sediment, the samples of 10 mm in diameter were made suitable for measuring the specific electrical resistivity (SER) via the eddy current method using a SIGMATEST 2.069 device (FOERSTER Int., Pittsburgh, USA) with a sensor of 8 mm in diameter. The uncertainty of measuring the SER was ±0.03 μΩ·cm. The measurements of microhardness (Hv) were performed using an HVS-1000 hardness tester (INNOVATEST Europe BV, Maastricht, Netherlands) at the load of 50 g. Hereafter, the magnitude of microhardness measured at the load of 50 g will be denoted as Hv_0.05_. The average uncertainty of measuring Hv_0.05_ was ±15 MPa.

To carry out the investigations of the microstructure of the alloys, a Leica^®^ DM IRM metallographic microscope (Leica Microsystems GmbH, Wetzlar, Germany), Jeol^®^ JSM-6490 scanning electron microscope (SEM, Jeol Ltd., Tokyo, Japan) with Oxford Instruments^®^ INCA 350 EDS microanalyzer (Oxford Instruments pls., Oxford, UK) and Jeol^®^ JEM-2100F transmission electron microscope (TEM, Jeol Ltd., Tokyo, Japan) were employed. The samples were subjected to mechanical grinding on sanding 6-H paper with the particle sizes of 63–80 μm and polishing in advance using diamond pastas with the particle sizes from 20 to 28 μm down to < 1 μm. The electrochemical polishing at the finishing stage was conducted in a CrO_3_+H_3_PO_4_ electrolyte (current 3 A, voltage 30 V, 1 min). The microstructure of the alloys was revealed via chemical etching in HF (15 mL) + HNO_3_ (10 mL) + glycerin (35 mL) solution. The mean etching time was 1 min. The volume fraction of the recrystallized microstructure *f_R_* and the average grain sizes *d* were determined via the grain intersection counting method using GoodGrains software (Russia, UNN). The average uncertainty of determining the magnitude of *f_R_* was ±1 vol.% and the average relative uncertainty of determining the average grain sizes was ~10% of its magnitude *d*.

To investigate the thermal stability of the microstructure, the samples were subjected to annealing in air ambient using an EKPS-10 furnace (Smolensk SKTB SPU JSC, Smolensk, Russia). The annealing was performed in two regimes: (a) 60 min annealing in the range from the RT up to 550 °C and (b) isothermal annealing at 300 °C or 1000 h. The precision of maintaining the temperature (T) in the furnace was 10 °C. The samples were placed in the furnace heated up to the required temperature in advance. Cooling the samples after annealing was performed in air.

## 3. Results

### 3.1. Investigation of the Alloys in the Initial State

**Figure 1:**
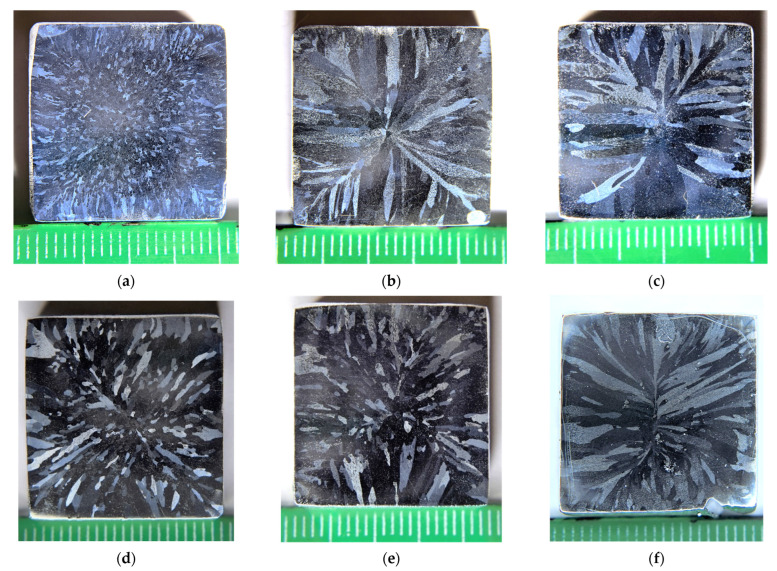
Macrostructure of the cast aluminum Alloys #1 (**a**), #2 (**b**), #3 (**c**), #4 (**d**), #5 (**e**) and #6 (**f**). The numbers in the figures correspond to the numbers of alloys in Table 1. Optical microscopy. Below each image in Figure 1, a ruler is presented. One scale unit corresponds to 1 mm.

Figure 1 presents the images of the microstructure of the aluminum alloys investigated. The specimens for investigations were taken from the lower parts of the bulks.

The macrostructure of the cast alloys comprises a mixture of columnar crystals at the edges of the samples and the equiaxial grains in the central parts of the bulks. The ratio of the areas occupied by the equiaxial grain zones and by the columnar crystal ones depends on the concentrations of the alloying elements. The largest area in the cross-section occupied by the equiaxial grains of 10–50 μm in size was observed for Alloy #1.

**Figure 2:**
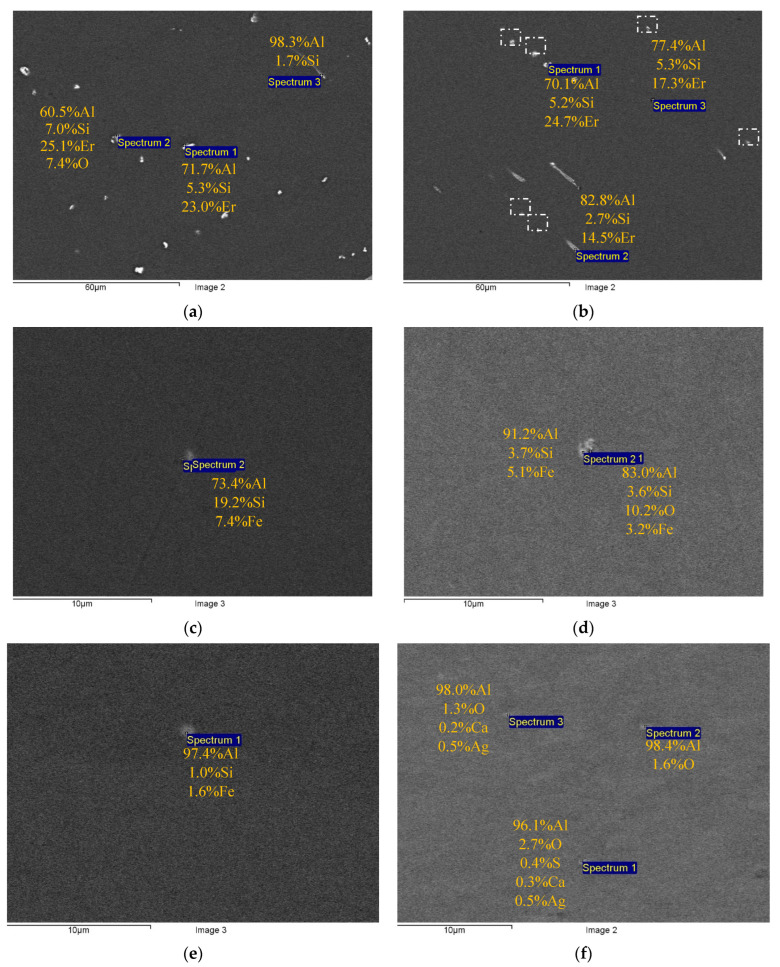
Comparative studies of the sizes and composition of the primary particles in the cast Alloys #1 (**a**), #2 (**b**), #3 (**c**), #4 (**d**), #5 (**e**) and #6 (**f**). SEM. BEC mode.

**Figure 3:**
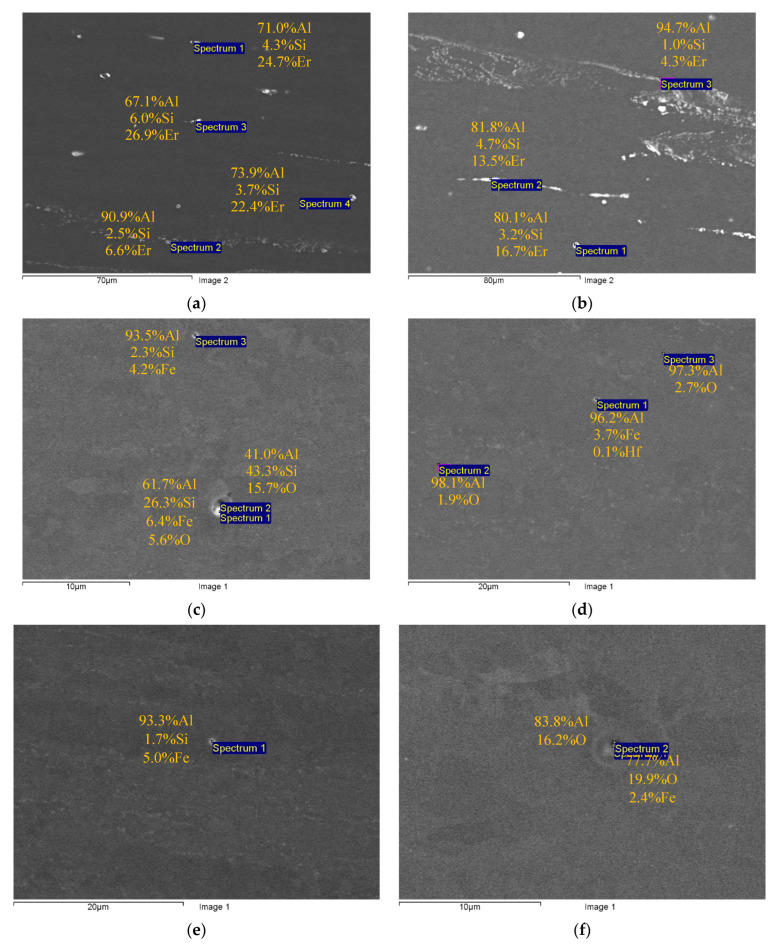
Comparative studies of the sizes and composition of the primary particles in the fine-grained Alloys #1 (**a**), #2 (**b**), #3 (**c**), #4(**d**), #5 (**e**) and #6 (**f**). SEM. BEC mode.

Figure 2 and Figure 3 present the results of SEM investigations of the composition and the character of distribution of the primary particles in the macrostructure of the cast (Figure 2) and deformed aluminum alloys (Figure 3). The samples, the surfaces of which were subjected to mechanical polishing, were used for investigations. The sample surfaces were not etched. The SEM investigations were carried out in the BEC regime (U = 20 kV, WD = 10, spot size = 40).

One can see the chains of light particles in the macrostructure of the cast Alloys #1 and #2. The large elongated particles are located preferentially at the dendrite boundaries. Inside the dendrites in Alloy #2, there are micrometer-sized particles, the shapes of which are close to the equiaxial ones. These particles are marked by a white dashed line in Figure 2b. The results of the EDS microanalysis show Al, Si and Er to be present in the composition of the particles in different proportions (Figure 2a,b). In Alloys #3–6, few round-shaped micron-sized light particles were observed (see Figure 2c–f). The EDS microanalysis revealed Fe, Si and sometimes oxygen in the composition of these particles. The Al-Fe-Si particles in Figure 2c–e are probably the β-Al_5_FeSi ore α-Al_8_Fe_2_Si ones [44,57,62,63,64]. No nucleation of primary particles enriched with Zr was found.

The composition of the primary particles in the fine-grained alloys was similar to the one of the particles in the cast alloys (Figure 2 and Figure 3). Note that in Alloy #4 (Al-0.25Zr-0.15Hf), particles with a low amount of Hf (<0.1 wt.%) were found.

The results of the microhardness and SER analyses derived from the alloys produced via induction casting are presented in Table 3. Alloys #1–3 alloyed with silicon had the maximum microhardness. The maximum values of SER were observed in the alloys with the addition of hafnium (Alloys #1, 4 and 5).

**Figure 4:**
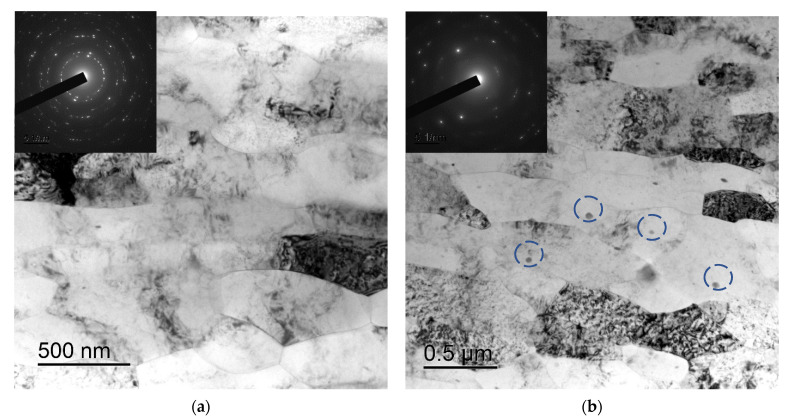
Microstructure of the fine-grained Alloys #4 (**a**) and #5 (**b**). TEM. The large particles formed during the crystallization of the alloy are marked by a dashed line in Figure 4b.

After severe plastic deformation using ECAP and rotary swaging, a strongly deformed microstructure with the sizes of fragments ~0.5 μm was formed in the samples of alloys (Figure 4). No essential differences in the microstructure of the fine-grained Alloys #1–6 were found. There were nano- and submicron-sized particles in the microstructure of the UFG alloys with increased contents of alloying elements (Zr + X ≥ 0.5 wt.%) (The largest particles are marked by the dashed line in Figure 4b.)

As one can see from Table 3, the hardness of the aluminum alloys increased ~1.6 times after SPD. The maximum values of microhardness (500–510 MPa) were observed for the fine-grained Alloys #1–2 having the highest value of Hv_0.05_ in the cast state as well (295–300 MPa) (Table 3). The magnitudes of SER of the aluminum alloys after SPD increased insufficiently (in 0.03–0.04 μΩ·cm) but this scale of variation or SER is comparable to the uncertainty of SR measurements via the eddy current method.

### 3.2. Effect of Annealing Temperature on the Properties of the Deformed Alloys

**Figure 5:**
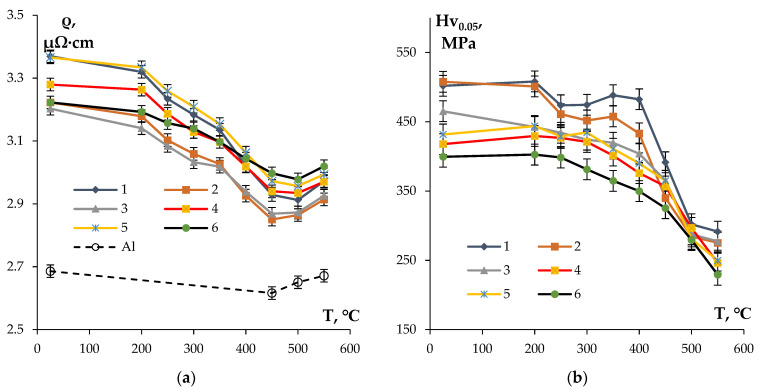
Dependencies of SER (**a**) and microhardness (**b**) of the fine-grained aluminum Alloys #1–6 on the temperature of 1 h annealing.

Figure 5 presents the dependencies of Hv_0.05_ and SER on the temperature of 1 h annealing of the fine-grained alloys investigated. The analysis of the curves ρ(T) shows that the magnitudes of SER decrease with increasing annealing temperature from 200 up to 450–500 °C for all of the samples. Obviously, this is related to the nucleation of the particles (see [33,34,35,41,45,69]). The Al-0.25%Zr-0.25%Hf alloy, whereby the magnitude of SER of 3.2 ± 0.05 μΩ·cm is achieved after annealing at 300 °C for 1 h, is characterized by the smallest intensity of SER variation in the temperature range 200–450 °C. The target level of SER of 3.0 μΩ·cm or less was achieved in all of the alloys after 1 h annealing at 450 °C. An increase in SER of ~ 0.1–0.15 μΩ·cm was observed at a further increase in temperature up to 550 °C, which, in our opinion, originates from the partial dissolving of large intermetallic particles of Al-Fe-Si (Figure 2 and Figure 3) and the increase in the Fe and Si contents in the solid solution. This suggestion was confirmed by the investigation of the temperature dependence of SER of pure aluminum, which was used for making the conductor aluminum alloys (Figure 5a).

**Figure 6:**
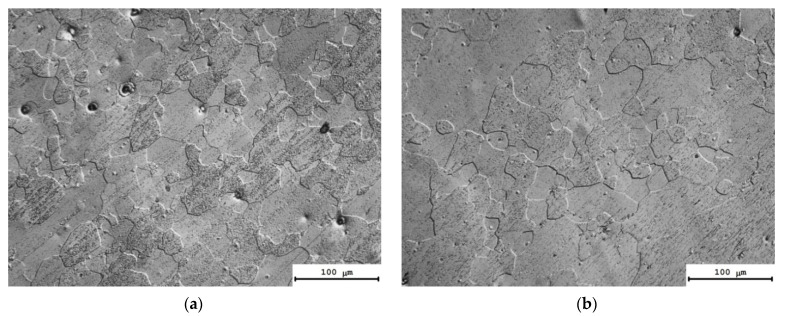

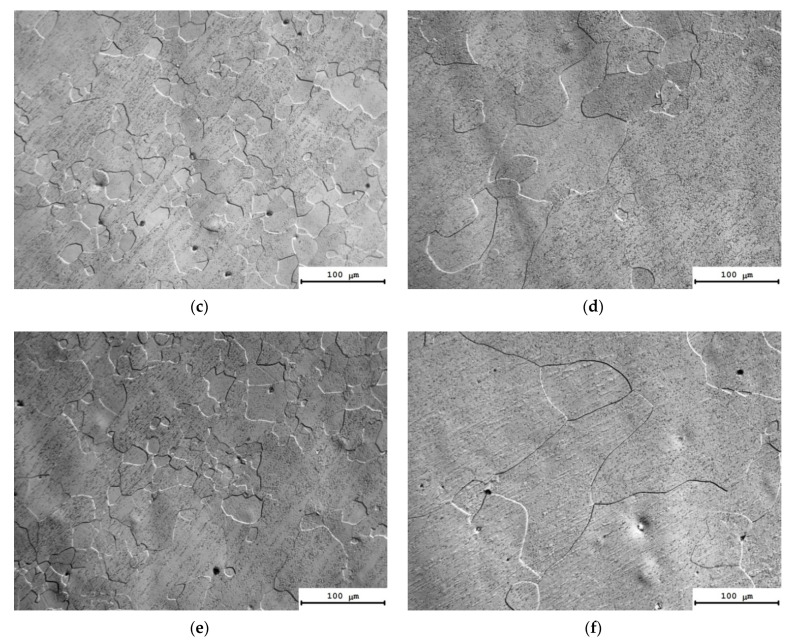
Microstructure of Alloys #1 (**a**), #2 (**b**), #3 (**c**), #4 (**d**), #5 (e) and #6 (**f**) after annealing at 550 °C for 1 h. Optical microscopy.

Figure 6 presents the results of investigations of the microstructure of the alloys after the annealing at 550 °C (1 h). All of the alloys have a fully recrystallized microstructure. One can see in Figure 6 that the mean grain sizes in the alloys after annealing at 550 °C (1 h) depend on the types and concentrations of the doping elements. The average sizes of the recrystallized grains in the Alloys #1, 2, 3 and 5 were 30–40 μm, whereas the grain sizes in the Alloy #4 (Figure 6d) and in the Alloy #6 (Figure 6f) exceeded 100 μm.

### 3.3. Effect of the Annealing Time on the Properties of Deformed Alloys

**Figure 7:**
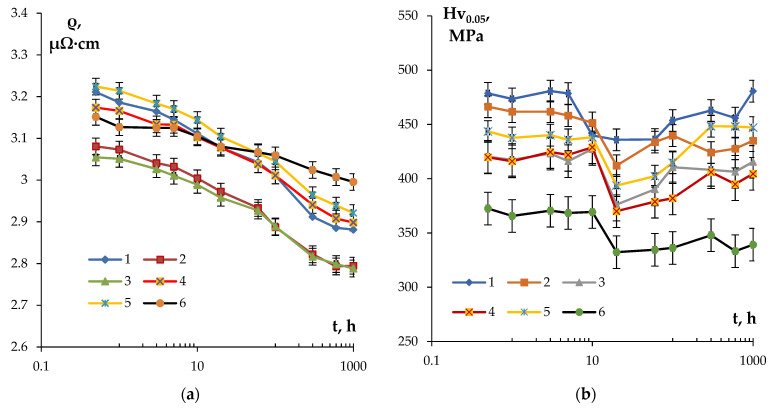
Dependencies of SER (**a**) and microhardness Hv_0.05_ (**b**) of the fine-grained aluminum Alloys #1–6 on the time of annealing at 300 °C.

Figure 7 presents the dependencies of SER and microhardness of the alloys on the time of annealing at 300 °C. The choice of the isothermic annealing temperature (300 °C) was motivated by the intention to minimize the grain growth intensity resulting in a decrease in the alloy microhardness and simultaneously ensuring a high intensity of the secondary particle nucleation. For the majority of alloys investigated, the degree of decomposition of the solid solution after 1 h annealing at 300 °C was close to 50%. This allows one to achieve the necessary (target) level of SER of the conductor aluminum alloy (3.0 μΩ·cm or less) whilst simultaneously ensuring its increased hardness.

The analysis of the curves ρ(t) presented in Figure 7a shows the SER of the fine-grained alloys to decrease exponentially with an increasing isothermic holding time. The most intensive decreasing of SER takes place in the first 20 h, and then the intensity of the SER decreasing drops considerably. The lowest intensity of the SER decrease (Δρ = 0.15 μΩ·cm) and, as a consequence, the highest values of SER in the annealed state (~ 3 μΩ·cm) were observed for Alloy #6. In the fine-grained Alloy #1, the scale of decrease in SER after annealing at 300 °C for 1000 h was Δρ = 0.33 μΩ·cm.

The dependence Hv_0.05_(t) had a more complex character. As one can see in Figure 7b, at small holding ties (t ≤ 20 h), the microhardness reduced in all fine-grained samples investigated, which probably originates from the recrystallization processes. The increase in the isothermic holding time leads to an increase in the microhardness, which originates from the nucleation of the secondary particles. The values of microhardness and SER in the annealed alloys are presented in Table 4. The maximum increase in the hardness during annealing was observed in Alloy #1; the hardness of Alloy #6 decreased continuously in the course of isothermic holding at 300 °C (Figure 7b).

**Figure 8:**
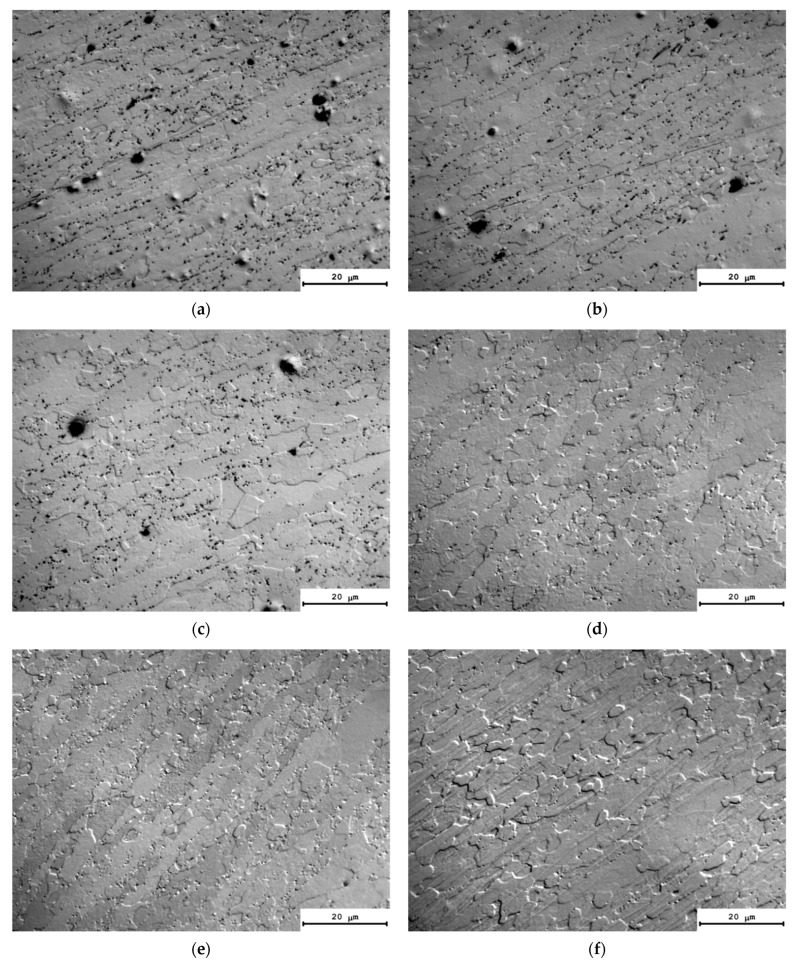
Microstructure of Alloys #1 (**a**), #2 (**b**), #3 (**c**), #4 (**d**), #5 (e) and #6 (**f**) after annealing at 300 °C for 1000 h. Optical microscopy.

The results of the investigations of the microstructure of the alloys after annealing at 300 °C, for 1000 h, are presented in Figure 8. The calculated values of the average grain sizes (*d*) and the volume fraction of the recrystallized microstructure (*f_R_*) are given in Table 4. The analysis of the results of investigations shows the long isothermic annealing at 300 °C not to result in a considerable increase in the average grain sizes. The volume fraction of the recrystallized microstructure was small enough and did not exceed 10%. The highest volume fraction of the recrystallized microstructure (~11–12%) and large grains were observed in Alloys #4 and #6 (Table 4).

**Figure 9:**
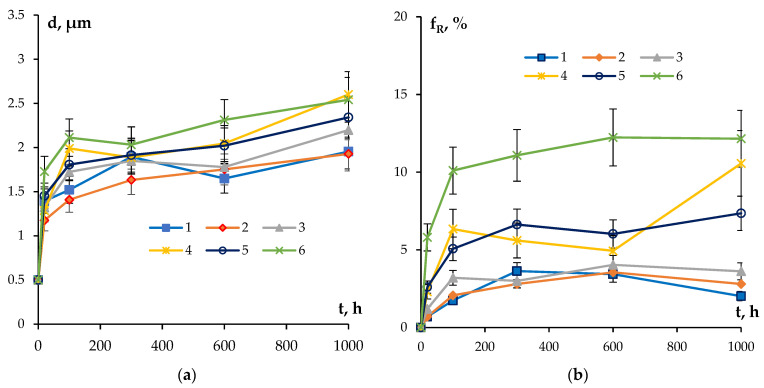
Dependencies of the average recrystallized grain sizes (**a**) and of the volume fraction of the recrystallized microstructures (**b**) on the time of annealing at T = 300 °C.

One can see a nucleation of the micrometer-size particles in the microstructure of the partly recrystallized Alloys #1–3 (Figure 8a–c). The secondary particles are located preferentially at the boundaries of the recrystallized grains. No line-wise nucleated micron-sized particles were found in the microstructure of partly recrystallized Alloys #4–6 but these ones are located preferentially at the recrystallized grain boundaries as well (Figure 8d–f). Figure 9 presents the dependencies of the average grain sizes and of the volume fraction of the recrystallized microstructure on the annealing time. One can see from these dependencies that the increase in the time of holding at 300 °C leads to an increase in the average grain sizes for all of the alloys. The volume fraction of the recrystallized microstructure also grows monotonously with an increasing annealing time. The minimum values of the volume fraction of the recrystallized microstructure were observed for Alloys #1–3 alloyed with silicon.

## 4. Discussion

### 4.1. Specific Electrical Resistivity. Kinetics of the Particle Nucleation

The theoretical values of SER (ρ_th_) of the alloys calculated under the assumption of the additive contributions of the alloying elements (Zr, Si, Er, Hf and Nb) into the SER magnitude of pure aluminum are given in Table 3. This calculation can be made according to the Matthiessen rule using the following formula:

(1) $$\rho_{th}=\rho_{Al}+\sum^{\;}K_{i}C_{i}$$

where $\rho_{Al}$ is the SER of pure aluminum (2.7 μΩ·cm), *K_i_* is the contribution of the *i*^th^ element to the SER of aluminum and *C_i_* is the concentration of this alloying element (in % at.). The reference data for *K_i_* were taken from [72].

As one can see from Table 3, the theoretical values ρ_th_ for Alloys #1–3 and #6 are greater than the measured SER ones (*ρ_exp_*). In our opinion, the small measured SER values are related to the partial decomposition of the solid solution in these alloys at the crystallization stage. For Alloys #4–5 of the Al-Zr-Hf system, the opposite situation was observed—the theoretical values *ρ_th_* were smaller than the measured SER ones (Table 3). In further analysis, the magnitude of *K_Hf_* was taken to be equal to 0.75 μΩ·cm/at.%.

Let us determine the dependence of the volume fraction of the nucleated secondary particles on the time and temperature of annealing *f_v_*(t,T). The procedure of calculation of the volume fraction *f_v_*(t, T) based on the analysis of the results of investigations of SER *ρ*(t,T) was described in [69,70]. According to [69], the relationship between the *f_v_* and the variation in SER (Δ*ρ*) can be represented in the following form: *f_v_*(t) = Δ*ρ*(*t*)/*K* = (*ρ_0_* − *ρ*(t))/K, where ρ_0_ is the initial SER value (Table 3), *ρ(t)* is the SER after annealing with the duration t and *K* is a constant describing the contribution of the alloying elements constituting the nucleated particles into the SER of the alloy.

The expression for the volume fraction of the secondary particles *f_v_* can be represented in the following form [71]:

*f_v_* = C_r_(t)/α (2)

where α is the parameter characterizing the fraction of the impurity atoms in the particle (for the Al_3_Zr particles, the magnitude of α = 1/4 [71]) and C_r_ is the concentration of alloying elements entering the secondary particle. The maximum volume fraction of the particles can be calculated via the formula *f_v0_* = (C_0_ − C^*^)/α where C_0_ is the initial concentration of the alloying elements in the crystal lattice and C^*^ is the solubility limit of the alloying elements at a given temperature. To simplify the analysis, according to [71], let us assume that C_0_ >> C^*^ (in the case of the solid solution of zirconium in aluminum at 300 °C, one can assume that C^*^→0). The results of calculations of f_v0_ are presented in Table 3.

**Figure 10:**
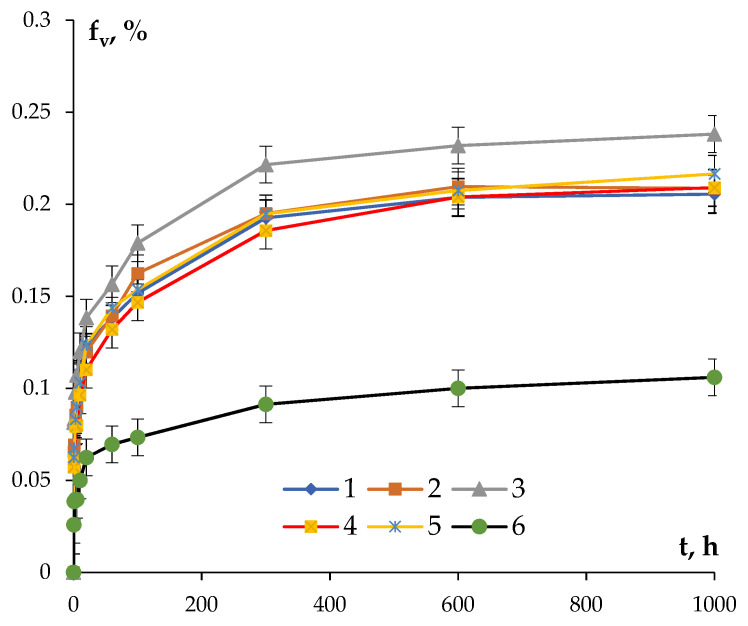
Dependencies of the volume fractions of the nucleated secondary particles in aluminum alloys at the time of annealing at 300 °C.

In this case, expression (2) can be transformed into the following form:

(3) $$f_{v}=f_{v_{0}}\left(\rho_{0}-\rho \left(t\right)\right)/(\rho_{0}-\rho_{min})$$

where *ρ_min_* is the minimum value of SER, which can be accepted to be equal to the SER of pure aluminum (2.7 μΩ·cm). The dependencies *f_v_*(t) calculated according to (3) at 300 °C are presented in Figure 10. The uncertainty of determining *f_v_* is governed, first of all, by the uncertainty of determining SER.

One can see in Figure 10 that the maximum volume fraction of the nucleated particles is characteristic for the Si-containing alloys. The minimum volume fraction of the nucleated particles was observed in Alloy #6. After holding for 600 h at 300 °C, the magnitudes of f_v_ tend to be the constant values in all of the alloys and remain almost constant with a further increase in the annealing time.

The dependence of the volume fraction of the secondary particles on the temperature and time of annealing can be described by the Johnson–Mehl–Avrami–Kolmogorov (JMAK) equation [70,71]:

*f_v_*(t, T) = *f*_*v*0_[1 − exp(−(t/τ)*^n^*)] (4)

where *τ* = *τ*_0_exp(*Q_1_*/*kT*) is the characteristic time of the diffusion process, *n* is the coefficient of the decomposition rate, *τ*_0_ is the pre-exponential factor, *Q_1_* is the effective activation energy of the particle nucleation process and *k* is the Boltzmann constant.

According to [70,71], the parameters *n* and *Q_1_* characterize the mechanisms of nucleation of the secondary particles. At the nucleation of the coherent particles in the fine-grained aluminum alloys of Al-(Sc,Zr), the relationship between the parameters of *n* and *Q_1_* and the mechanism of the solid solution decomposition can be described by the model [71]. In particular, the value of *n* = 1.5 corresponds to the case of homogeneous nucleation of the particles inside the crystal lattice, *n* = 0.75–1, and to the case of the particle nucleation at the grain boundaries or at the cores of the lattice dislocations. If at the annealing of a fine-grained material the recovery or recrystallization processes take place, the magnitude of coefficient *n* decreases down to 0.25–0.56 [71] (Table 5).

**Figure 11:**
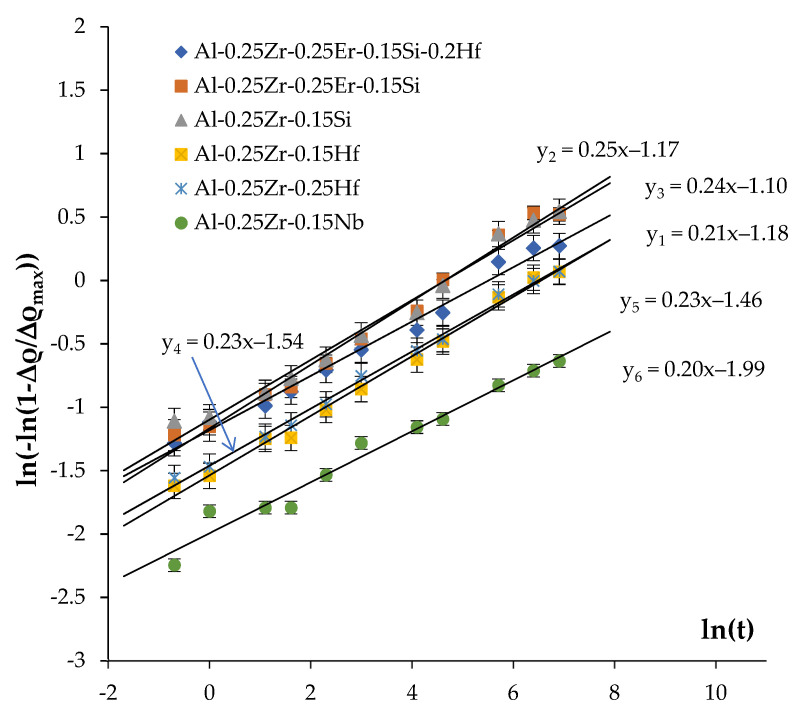
Analysis of the mechanisms of the secondary particle nucleation. Calculation of the coefficient *n* in the JMAK equation.

The values of *n* are determined from the measured curves *f_v_*(t,T) plotted in the double logarithmic axes ln(ln(1 − *f_v_*/*f_v0_*)) – ln(t) (Figure 11). In the case of the precipitation mechanism not changing, these dependencies should comprise straight lines, whereby the slopes of which give the magnitudes of the parameter *n* while the offset equals *n*·ln(τ).

As one can see in Figure 11, the values of the coefficient *n* fall into the range of 0.2–0.25 (Table 4). The confidence coefficient of the linear fit is R^2^ = 0.97–099. The result obtained evidences that the nucleation of secondary particles takes place preferentially at the cores of the lattice dislocations in the conditions of simultaneous recovery and recrystallization processes (see [71]). This conclusion agrees qualitatively with the results of microstructure investigations (Figure 8 and Figure 9, Table 4).

So far, one can conclude that the improved thermal stability of the fine-grained microstructure of the aluminum alloy investigated can be provided by the nucleation of secondary particles at the cores of lattice dislocations. Note that it is an important result from the viewpoint of applications, which allows for the ensuring of the stabilization of the nonequilibrium microstructure of the aluminum alloys at reduced annealing temperatures.

### 4.2. Investigation of Microstructure. Particle Growth during Annealing

As one can see from the experimental data presented (Figure 8), the grain growth with simultaneous nucleation and growth of the secondary particles takes place in the course of annealing (Figure 10). The particles nucleating during annealing prevent the migration of grain boundaries and provide the increased thermal stability of the nonequilibrium microstructure of the fine-grained aluminum alloys. According to [73], the relationship between the particle size *R*, the volume fraction of these ones *f_v_* and the average size of the recrystallized grains *d* is determined by the Zener equation:

*d* = α_1_*R*/*f_v_* (5)

where α_1_ is a coefficient depending on the particle shape (α = 4/3 for the spherical particles). It follows from Equation (5) that one has to ensure a high intensity of nucleation and a low growth rate of the particles in order to provide increased stability of the microstructure.

**Figure 12:**
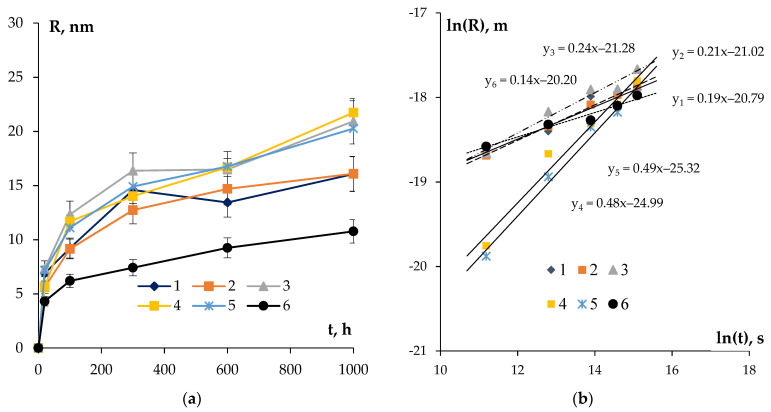
Dependencies of the particle sizes in the aluminum alloys on the time of annealing at T = 300 °C in the linear (**a**) and logarithmic (**b**) axes.

Comparing the results of the metallographic investigations, on the base of which *d* was determined (Figure 8a) and whereby the ones of the SER investigations allowed for the determining of f_v_ (Figure 10), let us calculate the size of the nucleated secondary particles *R* in the aluminum alloys. The curves *R*(t) at T = 300 °C are presented in Figure 12. The uncertainty of determining the magnitude of *R* is governed by the uncertainties of determining the magnitudes of SER and Hv.

One can see in Figure 12a a monotonous increase in the average particle sizes in the course of annealing at T = 300 °C to be observed in all of the alloys. All of the alloys had an approximate growth rate of the secondary particles except for Alloy #6 containing Nb in its chemical composition. On average, the particle sizes in the alloys increased 2–3 times from the initial values after 1000 h of annealing.

The growth of the secondary particles is known to be described by the following equation [73,74]:

(6) $$R^{m}-R_{0}^{m}=\xi D_{0}t\exp(-Q_{2}/kT)$$

where *R* and *R_0_* are the current and initial radii of the secondary particle, respectively, *m* is the growth rare coefficient, *Q_2_* is the activation energy of the secondary particle growth process, *ξ* is a numerical coefficient and *D_0_* is the pre-exponential factor in the diffusion equation. Using Equation (6), one can estimate the magnitude of the coefficient *m*, which depends on the dominating diffusion mechanism limiting the growth intensity of the secondary particles [70,71,74]. According to (6), the quantity 1/*m* corresponds to the slope of the curve *R*(t) in the logarithmic axes ln*R* – ln*t*.

Note that the magnitude of the coefficient m determines the dominating mechanism of the particle growth [70,71,74]. In the case of particle growth inside the crystal lattice of aluminum *m* = 3, at the grain boundaries and at the dislocation cores (in the case of a stale microstructure) *m* = 4 [70,71,74]. In the case of the microstructure of the aluminum alloy being unstable and the secondary particles nucleating at the cores of the lattice dislocations, the magnitude of the coefficient *m* varies from 6 to 12 [71,74].

The analysis of the data presented in Figure 12b shows the minimum values of the coefficient *n* to be observed for Alloys #4–5 of the system Al-0.25%Zr-Hf (Table 4). The result obtained evidences the Al_3_(Zr,Hf) particles, which nucleated first at the cores of the lattice dislocations (see Section 4.1) to grow further via volume diffusion. This can occur because of a simultaneous decrease in the density of the lattice dislocations as well as because of a large (as compared to Zr) intensity of diffusion of the Hf atoms in the crystal lattice of aluminum at 300 °C.

The fine-grained Nb-containing Alloy #6 has the highest value of the coefficient *n* = 6.9 among the alloys investigated (Table 4). The result obtained evidences the rates of nucleation and growth of the Al_3_(Zr, Nb) particles in this fine-grained alloy to be limited by the intensity of diffusion in the dislocation cores.

For the Si-containing fine-grained Alloys #1–3, the magnitude of the coefficient n ~ 4–5 probably points to the growth of the secondary particles at the grain boundaries (see [74]). This conclusion agrees well with the rapid nucleation and growth of the secondary particles in the Si-containing aluminum alloys described above.

Summarizing the results of the analysis performed, one can conclude that the nucleation of the secondary particles in the alloys investigated takes place at the cores of dislocations while the mechanism of their further growth depends on the type of the relationship of the volume, dislocation and grain boundary diffusion coefficients of the alloying elements in aluminum at a given annealing temperature as well as on the character of their spatial distribution (the uniform distribution inside the material bulk or formation of the grain boundary segregations).

### 4.3. Optimization of Microhardness and SER

First, the effect of doping elements on the properties of the aluminum alloy should be discussed, first of all—silicon and iron. The results of EDS microanalysis presented in Figure 2 and Figure 3 evidence the presence of silicon and iron in the composition of some primary particles. Iron is one of the unwanted impurities often present in aluminum alloys. It is known from the literature that the solubility of Fe in aluminum is very low (0.05% at 650 °C), and the primary β-Al_5_FeSi and/or α-Al_8_Fe_2_Si particles are formed during the crystallization of the aluminum Al-Si-Fe alloy (see [24,44,57,62]). Since silicon reduces the electrical conductivity of aluminum, the formation of the Al-Fe-Si particles will lead to an increase in the electrical conductivity of the aluminum alloy [24]. Since the Al-Fe-Si particles and other primary particles have large enough sizes while the volume fractions of these ones are small, we do not expect any essential effect of these ones on the strength or thermal stability of the fine-grained alloy.

**Figure 13:**
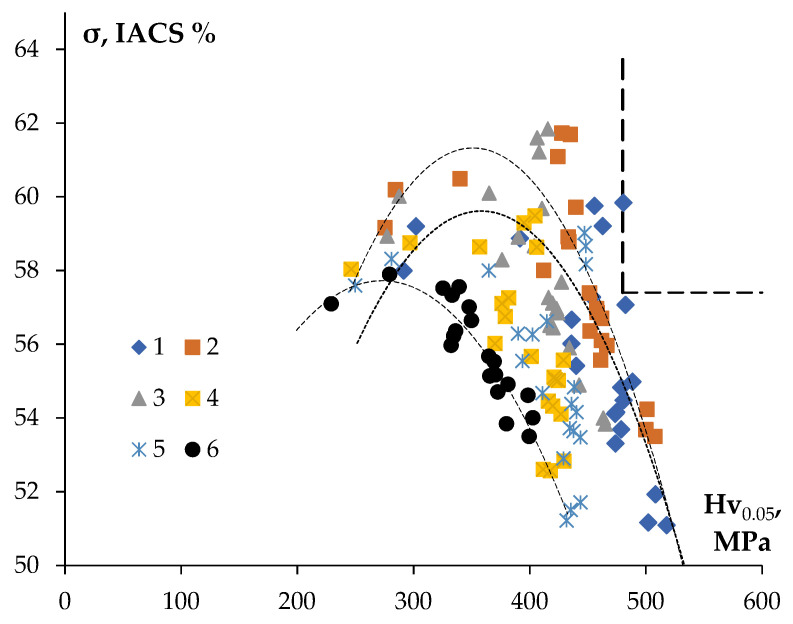
Dependence of the electrical conductivity (IACS) on the microhardness for the fine-grained aluminum alloys of Al-0.25Zr-(Si, Er, Hf and Nb).

Figure 13 shows the dependencies of the electrical conductivity (in IACS units) on the microhardness for the aluminum alloys investigated. This chart is a convenient tool for optimizing the thermal processing regimes of the aluminum alloys of Al-0.25Zr with different compositions of the alloying additives allowing for selecting the optimal relation between the hardness and SER.

As has been already mentioned above, the target value of SER, which is necessary to achieve in the conductor aluminum alloy, is 3.0 μΩ·cm, which corresponds to 57.4 IACS. The minim value of the ultimate strength for the new conductor alloy should not be less than that of alloy 01417 (σ_b_ ≥ 160 MPa). For the fine wires from the aluminum alloys, the microhardness Hv is related to the magnitude of the ultimate strength σ_b_ by the relation Hv = β·σ_b_, where β is a numerical coefficient depending on the structure state of the alloys [33,34]. For the fine-grained conductor alloys β = 1.43, and for the annealed aluminum alloys β = 3.45 [33,34]. The aluminum alloys investigated were in partly recrystallized states (Table 4) and the magnitude of the coefficient β for these ones can be accepted to be equal to ~3. So far, the minimum value of microhardness for the aluminum alloys investigated should be Hv ≥ 480 MPa. The ranges of parameters (the microhardness and the electrical conductivity), which the materials of a fine wire should fall into, are shown by a dashed line in the upper right corner of the chart “electrical conductivity—microhardness” in Figure 13.

The characteristics of Alloy #1 after annealing at 300 °C for 1000 h (59.8%IACS, Hv = 480 ± 15 MPa) satisfy this range of the values. This alloy has a uniform fine-grained structure with grain sizes ~ 2 μm (Table 4, Figure 8a). Additionally, the characteristics of Alloy #1 after annealing at 400 °C for 1 h (57.1%IACS, Hv = 482 MPa) are the closest to this range of parameters.

In our opinion, to improve the combination of properties of Alloy #1 further, one should optimize its chemical composition as well as optimize the casting regimes for Alloy #1; as one can see from Table 2, the formation of large primary particles took place during the crystallization of Alloy #1. These particles did not contribute to the strength and electrical conductivity of the alloy essentially. According to the results of the EDS microanalysis (Figure 2a and Figure 3a), there are considerable concentrations of silicon (Si) and erbium (Er) in the composition of the primary particles. No traces of Zr and Hf were found in the composition of the primary particles (Figure 2a and Figure 3a). The result obtained allows the concluding of the reduction in Si and Er concentrations and would allow for the avoiding of the formation of large primary particles during crystallization. The large primary particles can grow via coalescence at further annealing and reduce the intensity of the nucleation of the nanoparticles providing the increased strength and stability of the fine-grained aluminum alloy. Additionally, the large primary particles can lead to the failure of a fine wire during fabrication by rolling in rolls or drawing.

**Figure 14:**
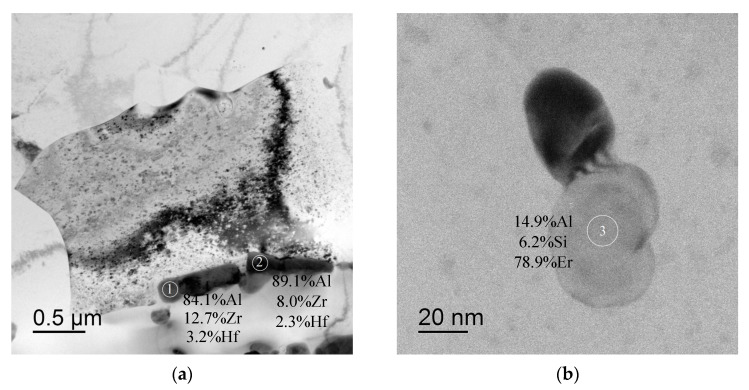
Structure of Alloy #1 after annealing at 300 °C for 1000 h: (**a**) Al_3_Zr particles along grain boundaries; (**b**) Al_3_(Er,Si) particles. The particle compositions are given in wt.%. TEM.

**Figure 15:**
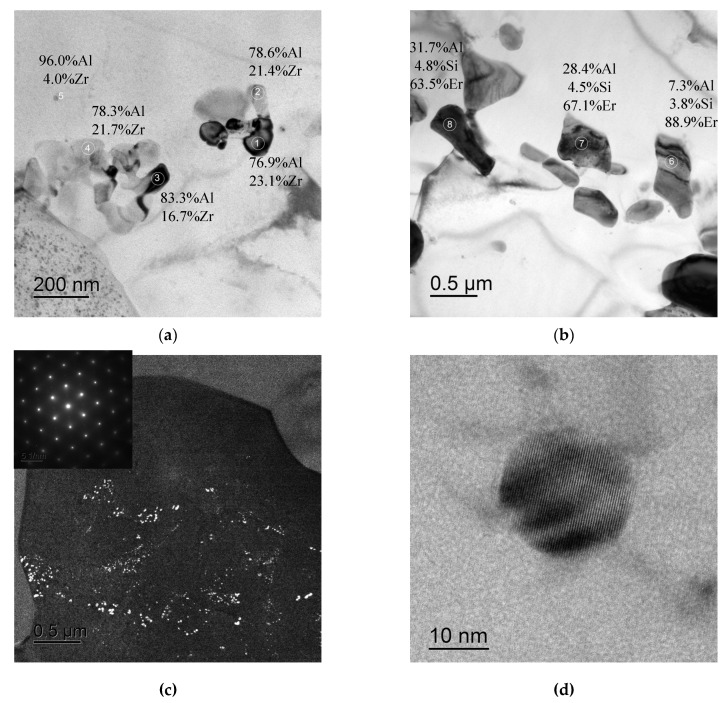
Structure of Alloy #2 after annealing at 300 °C for 1000 h. The Al_3_Zr particles (**a**) and Al_3_(Er,Si) ones (**b**). Figure (**c**) presents an image of the character of particle distribution inside the grain; Figure (**d**) presents an image of a small non-coherent Al_3_Zr particle. The particle compositions are given in wt.%. TEM.

In conclusion, it is worth noting that large non-coherent Al_3_Er particles, the composition of which includes Si and small non-coherent Al_3_(Zr, Hf) particles (Figure 14a,b), were observed in the structure of fine-grained Alloy #1 after annealing at 300 °C for 1000 h. No traces of intermittent decomposition of solid solution were observed. The particles were distributed uniformly enough inside the grains (Figure 14c). In Alloy #2, large and small Al_3_Zr particles (Figure 15a), as well as small submicron Al_3_Er particles with the addition of Si, were present after similar annealing at 300 °C for 1000 h (Figure 15b). The particles were located preferentially inside the grains (Figure 15c). Most particles, including the nanometer-sized ones, were non-coherent (Figure 15d). Note also that the secondary particles in Alloy #1 were distributed more uniformly than in Alloy #2.

So far, two types of particles are formed in Alloys #1 and #2 differing from each other by the presence (Alloy #1) or absence of Hf (Alloy #2) only: the Al_3_Zr/Al_3_(Zr,Hf) particles and the Al_3_Er ones with an addition of Si. Hafnium affects the ratio of hardness and electrical conductivity of the UFG Al-Zr alloy positively by reducing the Al_3_Zr particle sizes and providing a more uniform distribution of these ones inside the grains.

## 5. Conclusions

(1) The features of nucleation of the Al_3_(Zr, X) secondary particles during the annealing of fine-grained Al-0.25%Zr alloys with the addition of Er, Si, Hf and Nb were investigated. All of the alloys were found to preserve their fine-grained structure during annealing at 300 °C for 1000 h. The volume fraction of the recrystallized microstructure was small enough and did not exceed 10%; the average grain sizes were close to 2–2.5 μm. The mechanisms of nucleation of the secondary particles were identified via the analysis of the dependencies of the SER on the annealing time using the Jones–Mehl–Avrami–Kolmogorov (JMAK) equation. The magnitude of the coefficient *n* in the JMAK equation for the alloys investigated was shown to be close to 0.20–0.24, which corresponds to the case of nucleation of the secondary particles at the cores of dislocation in the conditions of simultaneous recovery and the recrystallization processes. Using the Zener equation, the dependence of the secondary particle sizes on the annealing time was determined. The secondary particle growth mechanism was shown to depend on the type of relationship between the diffusion coefficients for the volume, dislocation and grain boundary diffusion of the alloying elements in aluminum at a given annealing temperature, as well as on the character of their spatial distribution (uniform distribution in the volume of the material and the formation of grain boundary segregations). In the Hf- and Si-containing alloys, the secondary particle growth was controlled by the volume diffusion, while in the Nb-containing alloy it was controlled by diffusion via the cores of lattice dislocations.
(2) The effect of small (0.15–0.25%) additives of Er, Si, Hf and Nb on the thermal stability of the microstructure, SER and the microhardness of the conductor aluminum alloy Al-0.25%Zr were investigated. The Al-0.25%Zr-0.25%Er-0.20%Hf-0.15%Si alloy subjected to annealing at 300 °C for 1000 h has the optimal combination of microhardness and SER. The alloy after annealing has a uniform fine-grained structure; the average grain size was ~ 2 μm, the SER was 59.8%IACS and Hv = 480 ± 15 MPa. The high characteristics of this alloy (57.1%IACS, Hv = 482 MPa) can be ensured by annealing at 400 °C for 1 h. The optimal combination of hardness and electrical conductivity in the Al-0.25%Zr-0.25%Er-0.20%Hf-0.15%Si alloy is provided by means of the nucleation of two types of non-coherent particles—nano- and submicron-sized Al_3_(Zr,Hf) particles and small submicron-sized Al_3_Er ones with the addition of Si. The characteristics of the novel alloy allow for its efficient application in the aircraft building industry to replace the commercial eutectic alloys with increased contents of REEs and TMs.

## Figures and Tables

**Table 1:** Compositions of the aluminum alloys.

Alloy #	Contents of Alloying Elements in the Alloys, wt.%				
	Zr	Er	Si	Hf	Nb
1	0.25	0.25	0.15	0.2	-
2	0.25	0.25	0.15	-	-
3	0.25	-	0.15	-	-
4	0.25	-	-	0.15	-
5	0.25	-	-	0.25	-
6	0.25	-	-	-	0.15

**Table 2:** Casting regimes for the aluminum alloys.

Casting Regimes	Alloy #					
	1	2	3	4	5	6
Copper mistress, mm^3^	22 × 22 × 160					
Ceramic crucible volume, cm^3^	150					
Purging via argon prior to melting, cycles	3					
Purging via argon during heating, cycles	3					
Melt mixing	Induction					
Cooling down, s	250–50 under vibration					
Heating power, kW	4.5					
Time to the melting of the components	8 min 25 s	7 min 35 s	7 min 55 s	8 min 5 s	8 min 20 s	8 min 12 s
Melt temperature, °C	800					
Holding time prior to casting, min	20					
Casting temperature, °C	780					

**Table 3:** Microhardness and SER of the alloys in different states.

Characteristics	Alloy No.					
	1	2	3	4	5	6
	After casting					
Hv_0.05_, MPa	300 ± 10	295 ± 30	285 ± 10	250 ± 5	255 ± 10	250 ± 25
ρ, μΩ·cm	3.34 ± 0.06	3.19 ± 0.03	3.20 ± 0.01	3.25 ± 0.08	3.33 ± 0.05	3.18 ± 0.05
	After severe plastic deformation (ECAP + rotary swaging)					
Hv_0.05_, MPa	500 ± 15	510 ± 20	465 ± 15	420 ± 15	430 ± 15	400 ± 10
ρ, μΩ·cm	3.37 ± 0.03	3.22 ± 0.03	3.20 ± 0.02	3.28 ± 0.05	3.37 ± 0.04	3.22 ± 0.04
ρ_th_, μΩ·cm	3.47	3.45	3.23	3.15	3.16	3.43
f_v0_, %	0.28	0.26	0.29	0.32	0.33	0.26
	After SPD and annealing, 550 °C, 1 h					
Hv_0.05_, MPa	290 ± 5	275 ± 5	275 ± 5	245 ± 5	250 ± 5	230 ± 5
ρ, μΩ·cm	2.97 ± 0.05	2.91 ± 0.03	2.93 ± 0.01	2.97 ± 0.05	2.94 ± 0.04	3.02 ± 0.03
d, μm	14.4 ± 0.7	18.3 ±0.8	14.4 ± 0.4	24.2 ± 0.8	15.6 ± 0.7	31.3 ± 1.2

**Table 4:** Physical and mechanical properties, microstructure parameters, the values of coefficient *n* in the Johnson–Mehl–Avrami–Kolmogorov (JMAK) equation (see Equation (4) below, see [70,71]) and the values of coefficient *m* in the equation for the particle growth rate (see Equation (6) below) of fine-grained aluminum alloys after annealing at 300 °C (1000 h).

Alloy #	Experimental Data				Analysis of Results	
	H_V0.05_, MPa	ρ, μΩ·cm	*d*, μm	*f_R_*, %	*n*	*m*
1	480 ± 15	2.88 ± 0.04	2.0 ± 0.5	≤5	0.21	5.2
2	435 ± 10	2.79 ± 0.02	1.9 ± 0.4	≤5	0.25	4.8
3	415 ± 15	2.79 ± 0.04	2.2 ± 0.5	≤5	0.24	4.2
4	405 ± 10	2.90 ± 0.02	2.6 ± 0.6	11	0.23	2.1
5	445 ± 20	2.92 ± 0.06	2.3 ± 0.5	7	0.23	2.0
6	340 ± 10	3.00 ± 0.02	2.5 ± 0.6	12	0.20	6.9

**Table 5:** Parameters of JMAK equation (see (4)) and of the equation of particle growth (see (6)) for different diffusion mechanisms and driving forces of the decomposition of the solid solution [71].

Dominating Diffusion Mechanism ^1^	Driving Force of Decomposition of Solid Solution			
	Supersaturation of Solid Solution ΔC		Growth of Coherent Particles ~1/r	
	*n*	*m*	*n*	*m*
Volume diffusion (*Q_1_* = *Q_v_*)	1.5 (3/2)	2	1	3
Grain boundary diffusion (*Q_1_* = *Q_b_*)	1	3	0.75 (3/4)	4
Diffusion in the dislocation cores				
(a) Case of absence in recovery process (ρ_v_ = const) and in grain growth (*d* = const) *Q_1_* = *Q_d_*	1	3	0.75 (3/4)	4
(b) Case of recovery processes (ρ_v_ ≠ const), no grain growth (*d* = const) *Q_1_* = *Q_d_*	0.33 (1/3)	9	0.25 (1/4)	12
(c) Case of simultaneous recovery (ρ_v_ ≠ const) and grain growth ($d^{p}-d_{0}^{p}=At$)				
*p* = 2; *Q_1_* = (*Q_d_* + *Q_b_*)/2	0.67 (2/3)	4.5 (9/2)	0.5 (1/2)	6
*p* = 3; *Q*_1_ = (3*Q_d_* + 2*Q_b_*)/5	0.56 (5/9)	5.4 (27/5)	0.42 (5/12)	7.2 (36/5)
*p* = 4; *Q*_1_ = (2*Q_d_* + *Q_b_*)/3	0.5 (1/2)	6	0.38 (3/8)	8

^1^ *Q_v_*—activation energy of diffusion in crystal lattice, *Q_b_*—activation energy of grain boundary diffusion, *Q_d_*—activation energy of diffusion in dislocation cores, ρ_v_—density of lattice dislocation, *p,* and *A*—numerical coefficients in grain growth equation.

## Data Availability

Not applicable.
